# The relationship between perceived stigma and perceived stress in cognitive decline: a survey of persons with mild cognitive impairment and their caregivers

**DOI:** 10.3389/fpsyg.2023.1293284

**Published:** 2023-12-07

**Authors:** Alison Warren

**Affiliations:** The Department of Clinical Research and Leadership, George Washington University School of Medicine and Health Sciences, Washington, DC, United States

**Keywords:** stigma, stress, mild cognitive impairment, Alzheimer’s disease, caregivers, personhood

## Abstract

**Introduction:**

While Alzheimer’s disease and other causes of dementia have rapidly become a global health crisis with growing incidence that is unabated, the incidence of Mild Cognitive Impairment (MCI) far exceeds that of Alzheimer’s disease. Persons with MCI demonstrate some level of cognitive impairment, but daily functions remain intact and there is no certainty that they will develop dementia. Yet, the possibility conjures a considerable amount of fear and anxiety, further fueled by a vast array of misconceptions and stigma. The pervasive nature of this stigma permeates society and culture at many levels. Persons with MCI who are at higher risk for development of dementia may be especially vulnerable to fear and stigma associated with the diagnosis. Based on this premise, the primary aim of this study was to examine the relationship between perceived stigma and perceived stress in persons with MCI and their care partners, including the relationship between income and the study variables. The secondary aim was to examine the effect of a combined cognitive rehabilitation and wellness program on these perceptions.

**Methods:**

Thirty participants were recruited from Mayo Clinic’s Health Action to Benefit Independence and Thinking (HABIT) program. MCI (*n* = 15) and care partner (*n* = 15) participants completed the Stigma Impact Scale (SIS) and the Perceived Stress Scale (PSS) before and after the HABIT program.

**Results:**

Average SIS and PSS scores decreased in the MCI, care partner, and combined groups, both pre- and post-HABIT. Linear regression was used to assess the relationship between perceived stigma and stress, controlling for income. A significant relationship was found between perceived stigma and perceived stress both pre and post-HABIT.

**Discussion:**

The results suggest a relationship exists between perceived stigma and perceived stress in persons with MCI and their care partners, and an educational program such as HABIT may strengthen this relationship by informing participants of potential challenges that occur in cognitive decline. Understanding these relationships may provide an opportunity to provide tools for this vulnerable population.

## 1 Introduction

Alzheimer’s disease (AD) has garnered a great deal of much needed attention as a global health crisis desperately in need of treatment options, if not a cure. The merit of this focus is unquestionable, yet perhaps less discussed is the concomitant health crisis of Mild Cognitive Impairment (MCI). MCI describes a syndrome characterized by some signs of cognitive impairment beyond that of normal aging, but activities of daily function remain largely preserved (Gates et al., 2019). The prevalence of MCI far exceeds that of AD (Gates et al., 2019), but unlike AD, MCI carries a great deal of prognostic uncertainty which can serve as a major source of psychological distress (Beard and Neary, 2013; Bermejo-Pareja et al., 2016). Furthermore, persons with MCI are often treated as if they are categorically destined to develop dementia and become vulnerable to similar negative associations and prejudices associated with dementia (Sabat, 2006). The overall prevalence of MCI is 22.7%, which is more than double that of AD (11.3%; Gates et al., 2019; Rajan et al., 2021). Some cases of MCI will proceed to clinical dementia, although there appears to be high level of variability within the diverse subtypes of MCI (Mitchell and Shiri-Feshki, 2009; Bermejo-Pareja et al., 2016). Furthermore, while research indicates higher rates of progression to dementia in persons with MCI (Moreira et al., 2019), it is important to note that some never transition to dementia, and remarkably, some even revert to normal cognition (Iraniparast et al., 2022).

Alzheimer’s disease and related dementias (ADRD) are the unfortunate targets of stigma that creates psychological stress and distress for persons with these syndromes, as well as their loved ones (Mittelman and Batsch, 2012; Burgener and Buckwalter, 2018). The nature of this stigma has created a scenario in which persons with MCI who do not yet have the disease, and may *never* progress the disease, are subject to the same or similar dementia-related stigma (Morris et al., 2020). In addition to dementia-related stigma in those who do not have dementia, a considerable amount of fear and anxiety surrounding the potential for developing dementia exists in this population (Stites et al., 2017), the depth of consequences of which are still under exploration. Indeed, the effects of dementia-related stigma in persons with MCI may undermine their well-being (Stites et al., 2017) by producing far reaching negative physical and neuropsychological outcomes associated with perceptions of stigma and perceptions of stress. The quality of life in caregivers of persons with MCI is also markedly affected in several physical and psychological domains, as this is an underserved population that is not typically offered support due to the less severe diagnosis of MCI (Carlozzi et al., 2018). These stressors experienced by caregivers can significantly increase allostatic load and therefore the chronic stress response (Carlozzi et al., 2018). Furthermore, in persons with MCI, perceived stress has been associated with accelerated cognitive decline (Aggarwal et al., 2014) and may serve as a modifiable risk factor (Katz et al., 2016; Koyanagi et al., 2019). In “healthy” populations, lower socioeconomic status, including income, is associated with higher levels of stress, whereas higher income is associated with lower levels of stress and better coping skills (Schmitt et al., 2023). However, it is unknown whether higher income levels serve as a protective factor in levels of perceived stress or stigma in persons mild cognitive impairment or their caregivers.

Persons with MCI who fear the development of AD are not without cause. As the most common form of dementia, AD is characterized by progressive impairments in memory, executive functions, mood, language, activities of daily living, and ultimately functions necessary to sustain life (Moreira et al., 2019; Alzheimer’s Association, 2021). In addition to the physical and psychological difficulties, it is important to remember that this disease is more than just a medical issue, it is also a social issue (Swaffer, 2014), for persons living with, affected by, and at risk for developing AD are subject to numerous psychosocial threats to self and identity (Beard and Neary, 2013). Examples of such threats include, but are not limited to negative stereotypes, discriminatory behaviors, and social isolation (Low and Purwaningrum, 2020). These psychosocial threats are part of the larger macrocosm of stigma and are by no means exclusive to AD, as they also occur in preclinical presentations of cognitive decline such as MCI. Persons with MCI therefore not only endure the fear and uncertainty about further cognitive deterioration, but as targets of dementia-related stigma are also assumed incorrectly that further decline is inevitable and treated as such. Once diagnosed with MCI (or AD), the person is transformed into a patient with the disease (Stites et al., 2018), and it is this departure from person to patient that can foster negative stereotypes and stigma associated with this label.

Stigma is defined as “the co-occurrence of labeling, stereotyping, status loss, and discrimination in a context in which power is exercised” (Hatzenbuehler et al., 2021, p. 1). Stigma results in the perceptions, attitudes, and behaviors toward the stigmatized that are discounting, discrediting, and dehumanizing, resulting in a *spoiled identity* (Goffman, 1974; Sabat, 2006). Stigma and resulting spoiled identity threaten one’s humanity as they are viewed principally via their label and/or flaw, whereby they become devalued and spoiled in the eyes of others (Murphy et al., 2011). Dementia-related stigma is associated with negative psychosocial effects in persons with dementia and care partners (Low and Purwaningrum, 2020), and importantly, this stigma can be experienced in persons affected by MCI (Sabat, 2006; Morris et al., 2020) long before they develop dementia, if ever. What’s more, the effects of stigma and associated distress may be especially pronounced in persons with MCI due to their still intact insight. Considering the even greater prevalence of MCI and the immense toll of dementia-related stigma, this patient population is situated in a unique area of need and opportunity for intervention.

### 1.1 The contribution of diagnostic labels to stigma in persons with MCI

Dementia is a clinical syndrome which encompasses a broad spectrum of cognitive deficits that vary by etiology (Hemmy et al., 2020) and span a broad continuum of decline. While there are several different types of dementias, all with varied rates of progression, symptomology, trajectory, and prognosis, AD is the most common form (Hemmy et al., 2020). The development of AD is a slow, lengthy, progressive process that may begin up to 20 years before the onset of symptoms (Yue et al., 2021). The umbrella term “predementia” is often used to signify a continuum of stages of cognitive decline that precede frank dementia (including AD; Bermejo-Pareja et al., 2021).

MCI is typically considered a prodrome of dementia (Breton et al., 2019), which itself may be preceded by subjective cognitive changes. An individual may subjectively notice a deterioration of cognitive faculties who, upon objective measurement do not demonstrate any neuropsychological deficits (Yue et al., 2021). This subjective cognitive decline (SCD) may precede the transitional stage of mild cognitive impairment (MCI), in which cognitive impairment is more pronounced but does not meet functional criteria for dementia (Moreira et al., 2019). In contrast to AD, the daily functioning of persons with MCI is largely unaffected and independence is still preserved (Gates et al., 2019; Futschek et al., 2023). SCD and MCI are considered preclinical stages of ADRD (Futschek et al., 2023). This widespread assumption of MCI as a prodrome or preclinical stage of dementia may foster psychological stress (Sabat, 2006), despite the ambiguity of this assertion.

The considerable amount of variability and “predictive imprecision” in MCI could be considered to be quite positive, however, the fear associated with this uncertainty for a person with MCI or SCD may pose a significant threat to one’s psychological well-being (Beard and Neary, 2013). In addition to the fear of progression, it is common for those diagnosed with MCI to fear negative reactions in others (Sabat, 2006; Morris et al., 2020). Furthermore, the label of MCI can contribute to spoiled identity as the diagnosis becomes attached to the person as the main construct of their self-attributes and social identity (Sabat, 2006). In this way, the diagnostic label of MCI (as well as AD) changes the social dynamics between the person with MCI and healthy others, creating a negative “us/them” dynamic (Morris et al., 2020). A person diagnosed with MCI, therefore, is then viewed as a mildly defective patient who will become increasingly burdensome over time (Sabat, 2006).

Beard and Neary (2013) conducted a qualitative study involving interviews with 18 individuals with MCI which uncovered a few main themes among participants’ perceptions, including uncertainty concerning definitions of memory loss, MCI, and AD, in addition to confusion surrounding the boundaries of normal aging and dementia. They concluded that the perceptions of MCI patients mirror the nosological discrepancies, and further uncovers the social and psychological tension in these individuals (Beard and Neary, 2013). A more recent study by Morris et al. (2020) collected data from 10 MCI-care partner dyads based on focus group discussions to gain further insight into their feelings and perceptions about an MCI diagnosis. Their results revealed overarching themes driving diagnostic evaluation of (1) “presence of threat” and (2) attempts to “minimize the threat” by the dyads. They further identified subthemes of the “presence of threat” including fear of stigma and emotional reactions ties to the MCI diagnosis. Subthemes of attempts to “minimize the threat” of MCI included use of language, information sharing and withholding, and the use of social support (Morris et al., 2020). Their results further support the uncertainty and fear associated with the diagnosis of MCI, along with coping strategies used by MCI-care partner dyads (Morris et al., 2020).

Certainly, early prevention is ubiquitously ideal, but MCI may represent a stage of interventional opportunity. Indeed, the heterogeneity of MCI progression and prognosis warrants careful consideration of its diagnostic ramifications, as a “diagnosis of MCI can be both stigmatizing and anxiety-provoking” (Breton et al., 2019, p. 233). This fear, stigma, and anxiety alone may affect one’s prognosis.

#### 1.1.1 Exploring dementia-related stigma and its impact on persons with MCI

In order to appreciate the effect of dementia-related stigma in persons with MCI, it is important to understand the origins, manifestations, and consequences of stigma in persons with dementia that commonly transfer to persons with MCI. The many challenges brought forth by Alzheimer’s disease and related dementias (ADRD) have created a common narrative of this disease as an inevitable loss of self, a kind of death before death (Bitenc, 2020). As cognitive abilities continue to deteriorate, the person with dementia may be viewed as “not themselves anymore,” “less than,” or “the other.” The behaviors that follow these perceptions may contribute to the threats to self that are experienced by person with dementia. Such threats include harmful attitudes and behaviors, depersonalization, and stigma (Cahill, 2021). The act of stigmatization is actively discrediting, and thus strips an individual of value, effectively reducing them to one who is tainted and discounted (Goffman, 1974; Pachankis et al., 2018). There is a considerable amount of fear of developing ADRD that spans most nations and cultures (Rosin et al., 2020; Cahill, 2021) and is one of the most feared conditions in late life (Bystad et al., 2016; Cahill, 2021). The powerful impact of stigma occurs at many levels that range from self-stigma, interpersonal stigma, and structural stigma, all of which tend to foster social exclusion (Hatzenbuehler and Link, 2014). The subjective experience of stigma may include both the experience and perception of stigma; the distinction connoting that experienced stigma includes the stigmatizing behaviors of others, and perceived stigma denotes the perception of others’ behaviors and reactions by the stigmatized individual (Burgener et al., 2015).

Indeed, AD stigma is pervasive in the general population, family members, caregivers, and even in physicians (Bacsu et al., 2020; Sarmento, 2020) and is not limited to persons with AD. AD stigma affects persons with MCI, who may never develop AD, and includes healthcare encounters, which is compounded by the additional challenge of differentiating MCI from early-stage dementia for many providers (Beard and Neary, 2013). Sarmento (2020) distributed a multiple-choice questionnaire to neurologists, neurology residents, and neurology staff about their general knowledge, opinions, feelings and perceptions, and prejudices. The results demonstrated a significant level of stigma in all groups, especially non-neurologists (Sarmento, 2020). Many family physicians, who are often the first point of contact for persons with dementia or mild cognitive impairment (MCI), feel ill-equipped to provide care for this population (Bacsu et al., 2020). A 2015 survey of Canadian family physicians found that only 2 out of 5 felt properly educated to provide care for patients with cognitive decline, and this lack in education can lead to stigmatization that results in barriers in healthcare access, delayed diagnosis, and decreased quality of life (Bacsu et al., 2020).

The 2012 World Alzheimer’s Report highlighted the survey conducted by the Alzheimer’s Disease International of persons with dementia and family caregivers (*n* = 2,500) across 54 countries about their experience with stigma revealed that 75% of experienced stigma (Mittelman and Batsch, 2012). Sixty percent of respondents with dementia indicated that friends and family lost contact or avoided contact following their diagnosis, and both persons with dementia and caregivers avoided close relationships (Mittelman and Batsch, 2012). Furthermore, their survey data found that nearly 1 in 4 persons with dementia and 1 in 10 caregivers conceal the diagnosis of dementia due to stigma, and 40% of persons with dementia report being excluded from everyday life, further undermining psychosocial well-being and quality of life (Alzheimer’s Disease International, 2012; Mittelman and Batsch, 2012; Harper et al., 2019). The numerous inaccurate beliefs associated with AD reveal that stigma exceeds the diagnosis itself, as subjective experiences of persons with dementia are also assumed and discredited (Ashworth, 2020), likening the person with dementia to “a physical body left to be managed”; “incompetent”; “burdensome”; the “living dead” (Rosin et al., 2020). These stereotypes are typically born from the latest stages of the disease when a person is significantly impaired and dependent on others for care, yet beget behaviors which assume loss of competence, identity, and autonomy even in earlier stages the disease (Stites et al., 2018), or before disease is even evident to develop in MCI (Sabat, 2006). Such behaviors also affect how people with dementia perceive themselves. Ashworth (2020) sought to explore the perceptions of stigma in persons with early and late-onset AD by administering questionnaires (i.e., the Stigma Impact Scale) and semi-structured interviews. Of note, the 14 participants who participated in the more in-depth interview revealed higher levels of perceived stigma than the questionnaires, underscoring the nuances of stigmatized experiences such as feeling stupid, negative perceptions of others’ reactions, and altered relationships. The open-ended questions in the interview contributed to increased openness and therefore experiential details of persons with dementia and their caregivers, although both measures supported the existence of stigma in the dyads (Ashworth, 2020).

Burgener and Buckwalter (2018) examined stigma in persons with early-stage dementia and found that 42% of participants avoided disclosure of their diagnosis due to fear of consequences related to being stigmatized. This same study also highlighted the negative impact of stigma on quality of life for persons with dementia and caregivers, including but not limited to significant effects on anxiety, depression, personal control, self-esteem, physical health, activity participation, and social support (Burgener and Buckwalter, 2018). A longitudinal study by Burgener et al. (2015) examined perceived stigma (using the modified Stigma Impact Scale) in 50 persons with dementia and 47 corresponding caregivers at 4 time points over 18 months. In contrast to their hypothesis that perceived stigma would decline over the 18 months, perceived stigma remained significant and stable for the first year of study, only showing signs of abatement at the 18-month mark (Burgener et al., 2015). As they noted, the very stability of perceived stigma in persons with dementia merits earlier intervention, as is intended in this study in the earlier stage of mild cognitive impairment.

#### 1.1.2 The impact of dementia-related stigma on psychosocial well-being

One of the many challenges of measuring perceived stigma in persons with ADRD and some types of MCI is underscored by the variable deterioration of cognitive abilities. The decline in episodic memory may create difficulty in ascertaining the accuracy of self-reports, which may vary due to a multitude of factors observed in any participant independent of cognitive status, such as time of day, sleep status, and amount of time since their last meal, but may be more prominent in those who are cognitively impaired. Deterioration of language faculties may also create difficulty in their expression of stigma-related stress. The preservation of rich emotion, emotional memory, and maintained implicit memory (Sabat, 2006, Klein-Koerkamp et al., 2012; Fredericks et al., 2018) create a common scenario whereby persons with dementia feel the psychological effects of stigma but have difficulty recalling specific details to explain their experience and feelings.

The stigma associated with the label of AD, including in those with MCI who live in fear of AD, can have negative social consequences and alter self-perception relating to self-worth and competence (Stites et al., 2017; Stites et al., 2018). These negative outcomes are consistent with formal measures of stigma that reflect feelings of internalized shame, social rejection, and social isolation (Burgener and Berger, 2008; Harper et al., 2019). Stites et al. (2017) explored the impact of how the awareness of a diagnostic label impacted quality of life in persons with MCI (*n* = 92), mild AD (*n* = 68), and normal cognition (*n* = 99). They found that compared to participants who were unaware (anosognosia), persons with MCI and AD who were aware of their diagnosis reported lower scores on outcome measures including satisfaction with daily life, basic functioning, physical wellbeing. Additionally, their results demonstrated that those who expected their condition to worsen reported greater depression, higher levels of stress, lowering quality of life, and greater cognitive difficulties (Stites et al., 2017). These results underscore the increased vulnerability of persons with MCI and early AD who maintain awareness and insight to the negative effects of stigma.

Terminology can further contribute to stereotypes and the deleterious behaviors of malignant social psychology. *Malignant social psychology* is a term coined by Kitwood (1993) to refer to a collection of dysfunctional, yet innocent or unintentional behaviors that result in depersonalizing treatment toward persons diagnosed with dementia (Kitwood, 1993; Sabat, 2012). They include treachery, disempowerment, infantilization, condemnation, intimidation, stigmatization, outpacing, invalidation, banishment, and objectification (Kitwood, 1993). Associated behaviors may include using forms of deception with the intent to increase compliance, distract, or manipulate (treachery); now allowing a person to utilize their preserved abilities or failing to aid in completion of initiated tasks (disempowerment); treating a person patronizingly as if they are a child (infantilization); using threats or power to induce fear (intimidation); using a label or diagnosis as the basis for a person’s feelings or behavior (labeling); treating a person as an outcast (stigmatization); interacting with a person at a rate that is too fast for a person to understand or pressuring them to perform more rapidly than they can bear (outpacing); failing to acknowledge one’s subjective reality and feelings (invalidation); physically or psychology excluding a person (banishment); treating a person as an object rather than a sentient being (objectification; Kitwood, 2019). These negative social experiences can result in feelings of lowered self-worth and a diminished sense of self (Burgener and Berger, 2008).

Persons with dementia and persons with MCI are often the targets of the demeaning communications and behaviors of malignant social psychology, as well as stigmatization (Burgener and Berger, 2008; Burgener et al., 2015; Sabat, 2019; Morris et al., 2020). Sabat (2019) explored common examples of malignant social psychology in his manuscript of case reports and professional accounts as a psychology professor at Georgetown University. He illustrated a poignant example of two emergency medical technicians (EMT) who encountered a man diagnosed with AD with an injury that would normally necessitate an immediate mental status exam. However, the fellow EMT instructed him not to bother since the patient had AD and “would not know anything anyway,” which was said aloud in earshot of the patient, illustrating examples of the disparaging and dehumanizing behaviors of malignant social psychology (Sabat, 2019).

Notably, even the term “dementia” denotes a removal of the mind from its Latin roots, lending to the depiction that one who is “demented” has suffered a loss of mind and self (Halewood, 2016). On the contrary, evidence supports the existence of retained awareness and implicit memory in persons with dementia (Sabat, 2006; Burgener and Berger, 2008; Warren, 2021). In fact, awareness of persons with dementia persists even into the late stages of the disease, without any association between discrepancy scores (measure of awareness) and Mini-Mental Status Examination (MMSE) scores over time (Clare and Wilson, 2006; Burgener and Berger, 2008). Our language, behavior, and interactions therefore, all play key roles in the fate of persons with dementia; whether they live well or become social outcasts (Cahill, 2021).

The popular stereotypes of dementia do not accurately reflect the actual symptoms or subjective experiences in persons with cognitive decline (Zimmermann, 2017; Rosin et al., 2020), yet are widespread. The misperceptions resulting from such stigma are pervasive and affect both the person living with dementia and MCI as well as their caregivers. In fact, many persist in realms of popular, scholarly, and medical depictions of persons with dementia, such as the portrayal of persons with dementia as zombies, which in turn perpetuate fear and dehumanizing behaviors toward persons with dementia (Ashworth, 2020; Thornber, 2020). Despite a wealth of evidence to the contrary, the myths that fuel stigma (i.e., the person you knew will disappear, persons with dementia become like children, persons with dementia cannot have insight into their condition) permeate society, media, and medicine (Ashworth, 2020). Thus, stigma acts akin to a caustic solvent, eluting away dignity and humanity, and further fuel the fear experienced by persons with MCI. Media further contribute to public stigma in their portrayals of end-stage persons with dementia as looking lost, scared, and infantilized (Rosin et al., 2020). What’s more, AD advocacy groups who wish to reduce stigma, still rely on a certain amount of fear to increase donations, a counterintuitive but successful strategy well-supported by the literature (Rosin et al., 2020). However, the depictions in media do not accurately reflect persons with dementia or their caregivers. Zimmermann (2017) conducted a review of memoirs of persons with dementia and caregivers from the early 1990s to 2017 and found their sentiments and self-perceptions to be in direct opposition to media portrayals. The memoirs and autobiographies demonstrated that, despite public perception, these dyads maintain a strong desire for social interaction (Zimmermann, 2017; Rosin et al., 2020).

The stigma experienced by family members of persons with dementia and MCI may have a bi-directional impact. A study by Heinik et al. (2012) examined the relationship between caregiver stigma and caregiver burden in 185 caregivers for persons with AD. They found that caregiver stigma significantly increases caregiver burden, prevents caregivers from seeking services to reduce burden, and demonstrated major contributing factors of shame and decreased involvement of caregiving (Heinik et al., 2012). In this way, both persons with dementia and caregivers suffer isolation and increased stress and burden. Furthermore, caregivers of persons with dementia hold a belief that society does not want to hear about nor engage with persons with dementia, further exacerbating feelings of isolation, lack of support, and desperation (Kane et al., 2020). An earlier study by Burgener and Dickerson-Putman (1999) found consistency in the perceptions of persons with dementia and caregiver’s behavior, such as caregiver imposing restrictions on activities leading to a feeling of a loss of autonomy and meaningful activity. Most unfortunately, altered behaviors of caregivers and the public that are born of these misperceptions create frequent situations in which persons with dementia are not only isolated, but even “abandoned, believed devoid of humanity and personhood long before they actually die” (Thornber, 2020, p. 184). Stigma then, in persons with dementia and MCI, is the proverbial double-edged sword – creating a situation in which a person feels compelled to recoil into isolation while society concomitantly turns them away. To further compound this matter, persons with MCI may encounter additional harm could conceivably impede their prognosis, as social isolation itself is a risk factor for dementia (Dukelow et al., 2023).

#### 1.1.3 Stigma, stress, and distress

Many, if not arguably all, diseases may conjure a certain amount of fear and stress in an individual. Indeed, experiencing any physical and psychological illness, along with associated perspectives regarding mortality and quality of life, can create stress and distress in an individual. Due in part to the multitude of misattributions and stigma associated with dementia, cognitive decline of any kind is positioned uniquely among disease states, and MCI especially so because they do not and may never have dementia. Nevertheless, the fear, stress, and stigma associated with dementia is present in persons with MCI. This is, in part, due to the association of the loss of self that is attached to the diagnosis of cognitive impairment (Ashworth, 2020), which when formally diagnosed, connotes an irreversible loss of self (Halewood, 2016). The very uncertainty about *when or if* one will develop and succumb to cognitive decline may cause significant psychological distress in this regard (Rosin et al., 2020), especially those who are at higher risk such as the case of MCI. The late author, Terry Pratchett, captured this sentiment well when, upon learning of his diagnosis of AD, said it was as if he had two diseases, “one was Alzheimer’s and the other was knowing I had Alzheimer’s” (Pratchett, 2015, p. 1). A small study (*N* = 12) by Lingler et al. (2006) utilized semi-structured interviews to explore the subjective experience of patients with MCI. They found that fear and uncertainty was associated with not only symptom burden but also surrounding their prognosis (Lingler et al., 2006; Morris et al., 2020). In Morris et al.’s (2020) small focus group study of persons with MCI (*n* = 4) and care partners (*n* = 4), the participants expressed perceptions of stigma as well as a range of negative emotional reactions including vulnerability, powerlessness, and fear. Moreover, as the disease progresses, a decline in linguistic ability renders one insufficiently capable of expressing anything to the contrary. An illustrative corollary may be drawn from patients who experience locked-in syndrome, whereby a third party may perceive a loss of identity of the patient, but from the patient’s perspective they are still fully experiential but unable to communicate as much (Nizzi et al., 2018). In this light, no amount of scientific explanation, nor prose, can accurately depict the depth of conscious distress experienced by persons with dementia, particularly considering the subjectivity of outside evaluation of a cognitively compromised person. Taken together, these complexities emphatically accentuate the vulnerability to stigma in this patient population, including but not limited to that of stigma and stress.

The amount of stress experienced by persons who are the target of stigma-related attitudes and behaviors cannot be overstated. Stigma itself is a unique stressor and leads to psychological distress as the culmination of having a devalued social identity (Hatzenbuehler et al., 2009), including those suffering from dementia and MCI. The relationship between stigma, stress, and psychological distress has been well-supported (i.e., Hatzenbuehler et al., 2009; Hatzenbuehler and McLaughlin, 2014; Hatzenbuehler, 2016; Pachankis et al., 2018; McCleary-Gaddy et al., 2019). Hatzenbuehler et al. (2009) aimed to examine emotion regulation strategies related to stigma-related psychological distress. In their experience-sampling study, rumination and suppression occurred were more prevalent on days associated with stigma-related stressors. They also found that when stigma-related stressors were encountered, lesbian, gay, and bisexual respondents reported more isolation and less social support than African Americans respondents (Hatzenbuehler et al., 2009). In their second related study, they found that participants who ruminated following recall of a stigma-related event experienced prolonged distress on both implicit and explicit measures, supporting the stigma-distress relationship (Hatzenbuehler et al., 2009).

Many of the above studies have explored stigma related to weight, gender orientation, sexual orientation, race and ethnicity, and mental illness. However, dementia-related stigma is still in its infancy in the literature. The dearth of extant literature aimed at identifying the perspectives of persons with dementia limits not only the depth of knowledge, but also ideal intervention strategies, which may differ according to stage of decline (i.e., MCI vs. AD). Available literature is largely qualitative or anecdotal, the value of which notwithstanding, is juxtaposed with the paucity of quantitative controlled research (Burgener et al., 2015). One such longitudinal qualitative study by Burgener and Dickerson-Putman (1999) provided a glimpse of the patient perspective by asking early-stage persons with dementia to “describe themselves.” Negative self-attribution responses included “stupid,” “worthless,” and “in the way,” including those who were well-educated and successful (Burgener and Dickerson-Putman, 1999; Burgener et al., 2015). The extent to which the conscious experience of stigma and resulting internal distress contributes to adverse health outcomes has yet to be determined, the current limitation of which represents a dramatic shortcoming in the literature (Hatzenbuehler and Link, 2014).

Many strategies are utilized by stigmatized individuals to mediate this psychological distress of “mattering less” (Link and Hatzenbuehler, 2016), including maladaptive emotion regulation and coping strategies such as social isolation, rumination (Hatzenbuehler and Link, 2014; Pachankis et al., 2018), and suppression (Link and Hatzenbuehler, 2016). Positive coping strategies such as social support are also employed (Link and Hatzenbuehler, 2016), but again prove especially challenging for persons with dementia who are often isolated or relinquished to long-term care centers. It is noteworthy, therefore, that the adverse mental and physical effects of stigma in “healthy” (cognitively intact) MCI individuals are sufficiently severe to cause poor health outcomes, which demands an additional layer of urgency for stigmatized persons with dementia who are not afforded the same opportunities to voice their distress and trauma. Understanding the way in which stigma affects persons with, and at risk for, ADRD both psychologically and physiologically is imperative to their health, well-being, personhood, and quality of life. Not only does stigma cause psychological distress and poorer health outcomes across a multitude of stigma types (Richman and Hatzenbuehler, 2014; Pachankis et al., 2018), but in the MCI patient population it can also delay diagnosis and increase social isolation, thereby increasing the detriment to personhood, physical health, and well-being (Rosin et al., 2020).

#### 1.1.4 Stigma and the chronic stress response

Inflammation is involved in the development of AD, but it remains unclear if this is a cause or consequence, or vicious cycle involving both (Zhao et al., 2022). The aging process alone involves chronic low-grade inflammation as evidenced by elevated levels of C-reactive protein (CRP), and other proinflammatory cytokines such as tumor necrosis factor α (TNF-α) and interleukin-6 (IL-6; Zhao et al., 2022). Inflammation is part of any disease process including the cascade involving the chronic stress response. MCI progression to AD in response to stressful stimuli may be due to manifold consequences of HPA-axis dysfunction. Chronic stress of any origin can have detrimental effects on the mind, brain, and body. A causal relationship exists between chronic stress and HPA-axis dysregulation (Zhu et al., 2014). Cortisol released from the adrenal glands binds to glucocorticoid receptors that are widely distributed throughout the brain, and mineralocorticoid receptors that are predominantly localized to the hippocampus (Keller et al., 2017). Cortisol exerts a tonic influence via hippocampal mineralocorticoid receptors while pituitary feedback actions and amygdala activation are mediated by glucocorticoid receptors (Keller et al., 2017). Of note, the hippocampus is rich with cortisol receptors and thus vulnerable to the chronic stress response (McCleary-Gaddy et al., 2019; Hatzenbuehler et al., 2021), which is of particular concern in neurodegenerative disorders because it is a major target of neurodegeneration and memory decline, particularly the episodic memory decline observed in MCI and AD (Wang et al., 2022).

The dysregulated crosstalk between the brain and periphery has implicated the stress-responsive HPA-axis to disorders such as anxiety and depression (Keller et al., 2017), as well as AD etiology (Canet et al., 2019). In a study involving weight-related stigma, 170 participants consisting of overweight and lean groups were exposed to a laboratory stressor associated with weight-stigmatization scenario, and subsequently measured their stress response via cortisol reactivity (McCleary-Gaddy et al., 2019). They found that overweight participants in the stigmatizing condition demonstrated a blunted cortisol response consistent with chronic stress, whereas lean participants in the weight-stigmatizing condition demonstrated a rise in cortisol consistent with a normal stress response (McCleary-Gaddy et al., 2019).

Mineralocorticoid involvement in hippocampal and hemodynamic functions in the brain may become vulnerable targets for chronic stress (Keller et al., 2017). Furthermore, glucocorticoid over-secretion is highly toxic to limbic structures including the prefrontal cortex and hippocampus, and contributes to dysregulation of amyloid precursor protein processing, tau phosphorylation, neuroinflammation, oxidative stress, and excitotoxicity, all of which are involved in AD pathophysiology (Canet et al., 2019). In this way, a vicious cycle ensues between AD and HPA axis dysregulation, in which “AD induces the dysregulation of the HPA-axis, which in turn potentiates the pathology” (Canet et al., 2019, p. 2). Taken together, it is possible that HPA-axis dysregulation and AD pathology are in fact, bidirectional, and chronic stress therefore may serve as both precursor and result of this cascade of events.

Stigma of many types have been linked to HPA-axis dysregulation (McCleary-Gaddy et al., 2019; Mijas et al., 2021), although research correlating HPA dysregulation with MCI and AD stigma specifically is lacking. The evidence that is available in a variety of other types of stigmas supports the relationship between chronic stress associated with the experience of stigma and cascade of events leading to HPA-axis dysregulation, including chronic elevated or depressed levels of cortisol which result in immune suppression, altered glucose metabolism, and inflammation (Hatzenbuehler et al., 2021; Mijas et al., 2021). The chronic stress response therefore has far-reaching effects on several systems in the body, and neurobiological consequences likewise ensue. In fact, youths who experience stigma (i.e., gender, race, ethnicity) have been found to have smaller hippocampal volumes compared to nonstigmatized youths (Hatzenbuehler et al., 2021), in addition to blunted cortisol responses (Hatzenbuehler and McLaughlin, 2014). A study involving 74 lesbian, gay, and bisexual adults with the mean age of approximately 24 years from 24 states examined the relationship between perceived stigma and stress (Hatzenbuehler and McLaughlin, 2014). Results demonstrated that these young adults who were exposed to stigmatizing environments as adolescents demonstrated a blunted cortisol response, further supporting the biological relationship between stigma and the stress response (Hatzenbuehler and McLaughlin, 2014). The stress response and HPA-axis dysregulation caused by the experience of stigma therefore can influence both brain structure and function. Another study examining the neurological effects of stigma on developing brains examined 11,534 youths with a mean age of approximately 10 years old, compared participants who were exposed to stigma based on gender, race, and Latinx ethnicity to those where not exposed to stigma (Hatzenbuehler et al., 2021). They found via objective imaging measures that stigmatized youths had smaller hippocampal volume, in contrast to non-stigmatized youths who did not demonstrate hippocampal atrophy, supporting the specificity of stigma-related brain effects (Hatzenbuehler et al., 2021). Though these studies were not carried out with MCI or AD participants, the data do suggest a relationship between stigma, the chronic stress response, and objective brain changes. In totality, the available evidence suggests that stigma does not discriminate based on age, gender, race, or sexual orientation in its physical and psychological consequences.

#### 1.1.5 Neuropsychological ramifications of dementia-related stigma and stress

The fear associated with AD is not well understood and evidenced-based approaches to reduce this fear and stigma are paltry at best (Herrmann et al., 2018; Cahill, 2021). Nonetheless, stress, fear, and anxiety are experienced in those affected by AD and with MCI who are at a higher risk for AD, the very perception of which carries major ramifications that adversely affect quality of life (Riley et al., 2014). A pilot study by Smith et al. (2008) reported that persons with dementia had one or more symptoms of anxiety in 20% of those living in a dementia-specific assisted living center, and 100% of those living in a conventional assisted living center (Smith et al., 2008; Riley et al., 2014). Hynninen et al. (2012) assessed 169 patients with early mild dementia to examine the frequency and consequences of anxiety. They found 19.5% of patients had clinically significant anxiety, an additional 22.5% had subclinical anxiety, and approximately 50% had intermittent anxiety (Hynninen et al., 2012). They concluded that anxiety was correlated with depression, higher caregiver stress, and increased dementia-related impairment, but interestingly, anxiety was not associated with cognitive performance (Hynninen et al., 2012). To examine whether neuropsychiatric symptoms, including anxiety, affect global functioning in persons with dementia, Wadsworth et al. (2012) assessed 812 subjects (normal control, MCI, and AD) over a 3-year period. They found that symptoms of anxiety, apathy, and hallucinations were associated with increased global functional impairment and disease progression thusly (Wadsworth et al., 2012).

Chronic stress has marked deleterious effects on learning and memory. The negative relationship between cortisol, memory, and executive function has been well-established in the healthy adults and elderly (Gómez-Gallego and Gómez-García, 2019). However, these negative effects may actually lessen with disease progression, which would lend urgency to early prevention and intervention, especially in those with MCI. A study by Gómez-Gallego and Gómez-García (2018) measured salivary cortisol, anxiety, and memory in 46 mild-to-moderate AD patients compared to 52 controls and found that while healthy controls performed as expected (higher stress and anxiety was associated with poorer performance), the emotional memory of patients with AD was not related to the stress marker of salivary cortisol. Interestingly, in a follow-up study by the same researchers, they measured recall, verbal memory, and semantic memory in 80 patients with AD compared to 104 healthy controls (Gómez-Gallego and Gómez-García, 2019). They found similar results, in that, healthy controls demonstrated worse memory performance with elevated cortisol levels, but in AD patients the relationship between cortisol and memory is weakened, despite the AD group’s higher levels of cortisol (Gómez-Gallego and Gómez-García, 2019). It is possible, as they noted, that patients with AD have increased daily stressors inherent in the disease and daily living situation but lack the anticipatory stress response of an individual with normal cognition or MCI.

Perceived stigma may contribute to chronic stress and anxiety, especially earlier in the disease process of MCI, or even before any symptom onset. Perceived stigma is associated with amygdala reactivity owing to the fear and threat experienced by the person who feels stigmatized (Hatzenbuehler et al., 2021). Moreover, while it is well-established that stigma is associated with negative mental, psychosocial, and physical health outcomes (Rosin et al., 2020), very little research has focused on the influence of stigma on the rate of cognitive decline or the frequency of associated behavioral and psychological symptoms of dementia (BPSD). Kitwood’s (1993) writings highlight the ramifications of others’ behavior toward persons with dementia, such as stigmatization, that threaten personhood, including ‘malignant social psychology’ described earlier, such as internalized shame, lowered perceptions of self-worth, diminished sense of self, loss of personal control, and behavioral symptoms such as depression (Burgener and Berger, 2008; Burgener et al., 2015). Depression itself, which is part of the “dementing” process, may lead to feelings and behaviors of learned helplessness that can affect immune function, which in principle may suggest that patients with MCI may develop more problems in reaction to the diagnosis (Sabat, 2006). The neuropsychological ramifications of this conscious experience of stigma are extensive and have much yet to be uncovered.

The available evidence revealing the significant physical and neurological effects of stigma, however, is at once compelling and concerning, especially in the aging population who may be especially vulnerable to psychological and physiological insults. Investigation into the neuropsychological consequences of stigma, as it relates to the chronic stress response, psychopathology, and brain health could further elucidate the mechanisms by which stigma affects persons with MCI and dementia. Bridging this gap in literature may serve to enhance the education and policy implementations to mitigate stigma in this regard. In a similar vein, examining whether the physiological effects, along with the conscious experience of stigma create increased neuroinflammation and consequent acceleration in cognitive decline and increase BPSD may aid in advancement of the prevention and management of ADRD.

#### 1.1.6 The research problem

The negative psychosocial effects of stigma have been well-documented across a variety of stigma types, yet their overall impact on health remains underestimated (Pachankis et al., 2018). Indeed, despite the current global health crisis of Alzheimer’s disease and related dementias (ADRD; Olivari et al., 2020) and MCI (Rajan et al., 2021), the scope of these negative outcomes associated with stigma is still a nascent research focus. Understanding the full impact of the stigma associated with cognitive decline may not only provide a unique insight into the subjective experience of persons with MCI and dementia (Xanthopoulou and McCabe, 2019; Cahill, 2021), but it may also elucidate additional mechanism (s) by which the resulting distress contributes to the cognitive, mental, and physical deterioration inherent in this disease process (Xanthopoulou and McCabe, 2019). Currently, stigma-related research that explicitly evaluates dementia-related stigma is limited and evidenced-based interventions to address this stigma are likewise lacking (Herrmann et al., 2018). In addition to stigma targeted toward persons with and at risk for dementia, the scarcity of AD stigma research is also echoed in family stigma (Heinik et al., 2012). The literature to date reflects a preponderance of focus on caregivers’ perspective of stigma, rather than the perspective of persons with MCI and dementia, thereby limiting our knowledge of their subjective experience (Lion et al., 2021).

While the Alzheimer’s Society International and the World Health Organization have long acknowledged stigma as a having a central defining role in the experience of ADRD (Alzheimer’s Disease International, 2012), the way in which it may present, how best to study it, and avenues to combat it have been understudied (Harper et al., 2019). Furthermore, there is not yet a uniformly accepted “gold standard” measure for dementia-related stigma (Herrmann et al., 2018; Harper et al., 2019), nor a measure to assess changes over time (Herrmann et al., 2018), which represents key challenges when comparing studies. The paucity of literature, lack of assessment consensus, and limited evidenced-based interventions represent major gaps in dementia-related stigma research. The present study will use available validated measures to examine the relationship between dementia-related stigma, stress, and an integrative dementia intervention in persons with MCI and their care partners.

In addition to psychological consequences, the physiological effects of a variety of stigmas have also been explored. Relationships between stigma and the stress response are evidenced by dysregulation of the hypothalamic pituitary adrenal axis (HPA-axis; McCleary-Gaddy et al., 2019; Mijas et al., 2021) and upregulation of amygdala reactivity (Hatzenbuehler et al., 2021). However, the available literature does not address whether stigma induces the chronic stress response in persons with dementia specifically, nor if, or to what extent, this response contributes to the rate of cognitive decline and behavioral and psychological symptoms of dementia (BPSD), which are intimately related to levels of stress in persons with dementia and between the caregiver-patient dyad.

The present quantitative study aims to examine the relationship between stigma associated with ADRD and the chronic stress response in persons with mild cognitive impairment (MCI) as a preliminary step in understanding the presence and extent of experienced stress associated with dementia-related stigma. It will do so by assessing the perceived stigma in both persons with MCI and their primary caregivers because of the bi-directional relationship which exists between the stress of caregivers and the stress of those for whom care is provided. It will also assess the impact, if any, of income on levels of perceived stigma and perceived stress. Courtesy stigma experienced by caregivers may cause detrimental attitudes and behavior changes toward the persons with dementia (Van den Bossche and Schoenmakers, 2022), which in turn may exacerbate the conscious experience of stigma and stress in the person with dementia. Stress in a caregiver, through emotional and behavioral manifestations, may transfer to the person with cognitive impairment, and vice versa. The study will be conducted alongside a cognitive rehabilitation specialist-led treatment intervention. Perceived stigma and stress in patients with MCI and caregivers will be evaluated before and after the intervention. This study hypothesizes a correlation between dementia-related stigma and the chronic stress response, such that persons with MCI and caregivers who score high on perceived stigma will demonstrate commensurately high scores of perceived stress. This study also hypothesizes that income may serve as a protective factor in levels of perceived stigma and stress. It is possible that after an education focused program, dementia-related stigma may be reduced, which in turn may lead to reduced stress in both persons with dementia and caregiver.

Based on available literature, no other studies have specifically evaluated the relationship between MCI/ADRD stigma and the chronic stress response. This study will serve as a foundation for future research examining this relationship with more sophisticated biomarker analyses, as well as the possibility of accelerated cognitive decline due to the stigma-inducing stress response. The long-term goal of this research is to facilitate a means by which to dispel the widespread fear and misconceptions associated with this disease. In doing so, this knowledge may aid in the understanding of the unique distress experienced by persons with dementia and their caregivers that often remains unexpressed. Assessing this stigma-stress relationship in the earlier stage of MCI may provide the opportunity for early intervention to avoid future physical and psychological harm. Furthermore, the elucidation of the neuropsychological and physical stress induced by the experience of stigma may provide a clearer picture of the dynamic interplay of factors contributing to poor quality of life and cognitive decline to refine intervention methods.

The following study aimed to answer the following research questions:

1. How and to what extent do levels of perceived stigma affect levels of perceived stress?
2. How and to what extent does income level affect levels of perceived stigma and stress?
3. How and to what extent does the HABIT program affect levels of perceived stigma and stress?

### 1.2 Methods

A cross-sectional design with pre and post-intervention evaluation was chosen to evaluate the relationship between perceived stigma and perceived stress in persons with MCI and their care partners alongside the HABIT intervention.

#### 1.2.1 Participants

Following approval from the Harvard Committee on the Use of Human Subjects, along with permission from the Mayo Clinic, participation was offered to patients with MCI and their partners enrolled in the Mayo Clinic Jacksonville and Mayo Clinic Scottsdale Healthy Action to Benefit Independence and Thinking® (HABIT) program. Participants with early-stage dementia are also permitted to participate in HABIT with a partner if they have sufficient awareness and motivation to participate. Participants must be English speaking with sufficient reading and writing ability and served as their own controls. Mayo Clinic physicians obtain cognitive scores (MMSE equal to or greater than 24 and the Dementia Rating Scale (DRS) cutoff is 115) to determine eligibility in the program. Capacity to consent was also established by physicians at the Mayo Clinic prior to their acceptance to the HABIT program. There were no restrictions on gender, age, race, ethnicity, or sexual orientation for the purposes of this study. Those who elected to participate in this study underwent baseline measurement of perceived stigma and perceived stress before the program commencement. The same measures were repeated following the 10-day HABIT program on the last day.

#### 1.2.2 Intervention

The Healthy Action to Benefit Independence and Thinking (HABIT) program is a group-based cognitive rehabilitation and wellness program held four times per year and lasts for 10 days, for a total of 50 h of treatment (Locke et al., 2021). Admission to the HABIT program requires a diagnosis of MCI and allows a maximum of 10 dyads in Arizona and 14 dyads in Florida for each session. HABIT provides memory compensation training; cognitive exercise; yoga; didactic instruction in wellness behavior change including lifestyle modifications such as nutrition, sleep, emotion health for brain health, and future planning; and separate support groups for patients with MCI and their care partners (Chandler, 2017). The HABIT program is not specifically designed to alleviate perceived stigma or stress, but includes stress-related educational components such as tools for stress management, wellness education, readiness planning such as memory compensations, safety considerations, and advanced care planning, as well as group therapy sessions during which perceptions of stigma may be discussed if participants desire. Previous study evaluating the efficacy of the HABIT program demonstrated the ability of MCI patients to learn despite their memory impairment, as well as improvements in memory activities of daily living, sense of self-efficacy and quality of life in patients with MCI (Locke et al., 2021). For care partners, the HABIT program has been shown to improve mood, increase physical flexibility, and decrease anxiety (Locke et al., 2021). The participants in this study attended one full round (10 days) of the HABIT program.

#### 1.2.3 Measures

The following measures were chosen to evaluate levels of perceived stigma and perceived stress in persons with MCI and their care partners.

##### 1.2.3.1 Stigma impact scale modified for persons with dementia

The Stigma Impact Scale (SIS) is the most widely used, and only previously tested, measure of self-stigma in persons with dementia (Bhatt et al., 2021). It was originally developed by Fife and Wright (2000) as a 24-item scale to measure stigma in populations with HIV/AIDS, and later adapted by Burgener and Berger (2008) based on the Multidimensional Model of Perceived Stigma to utilize relevant wording for persons living with Alzheimer’s disease and Parkinson’s disease (Burgener and Berger, 2008; Weisman De Mamani et al., 2018; Bhatt et al., 2021). It contains three subscales: (1) social rejection, (2) social isolation, and (3) internalized shame (Burgener and Berger, 2008; Bhatt et al., 2021), and it represents two experiential domains including experiences of rejection and stigma, and social psychological feelings surrounding stigma (Burgener and Berger, 2008). Twenty-one items are rated on a 4-point Likert scale ranging from strongly agree (4) to strongly disagree (1), and a fifth option of “not applicable” (Burgener and Berger, 2008; Bhatt et al., 2021), with higher total scores indicating higher perceived stigma. The modified SIS captures the negative effects of stigma on self-esteem, as decreased levels of self-esteem are correlated with increased levels of internalized shame and social isolation in persons with dementia (Bhatt et al., 2021).

##### 1.2.3.2 Stigma impact scale modified for caregivers

Caregiver stigma is an important construct in the social psychological milieu of persons with dementia, and not only impacts the health and well-being of the caregiver, but also the person living with dementia. Therefore, it is also a major concern when considering the quality of life in the caregiver-person with dementia dyad. However, similar to perceived stigma in persons with dementia, affiliate stigma experienced by caregivers of persons with dementia is poorly represented in the literature (Chang et al., 2016). The above-mentioned modified SIS was adapted to the Caregiver Stigma Impact Scale by Liu et al. (2014) to assess perceived stigma in caregivers of persons with dementia (Liu et al., 2014; Weisman De Mamani et al., 2018). It consists of four subscales (Social Rejection, Financial Insecurity, Internalized Shame, Social Isolation) and 24 items using a 4-point Likert scale, with scores ranging from 0 to 96, higher scores indicate higher perceived stigma (Weisman De Mamani et al., 2018). Their reported internal reliability was excellent (Cronbach’s alpha α = 0.92; Liu et al., 2014; Weisman De Mamani et al., 2018).

##### 1.2.3.3 Perceived stress scale

Perceived stress refers to an individual’s cognitive appraisal of threats arising from a stressor, such that an individual’s resources are insufficient to meet the demands of the stressor (Lazarus and Folkman, 1984; Teresi et al., 2020). Therefore, the dynamics of stress, as an outcome of the transaction between individuals and their environment, vary in impact based, at least in part, to one’s perception of stress severity and their ability to cope with the stressor (Lazarus and Folkman, 1984; Deeken et al., 2018). The role of caregiver for persons with dementia is often assumed by family members, especially spouses (Deeken et al., 2018). The emotional strain of caregiving can result in stress that accumulates overtime into chronic stress, which is related to disease biomarkers, adverse physical and mental health outcomes, and increased mortality (Teresi et al., 2020). An inherent weakness in prior measures of global perceived stress did not account for this accumulated sensitivity to daily stressors (Cohen et al., 1983). Additionally, BPSD in persons with dementia may increase the likelihood that a caregiver will decide to institutionalize their family member with dementia, on account of caregiver distress, burden, and burnout (Pinyopornpanish et al., 2021). A recent cross-sectional study of 102 caregivers of patients with AD found that “caregiver burden is associated with patients’ neuropsychiatric symptoms indirectly through the caregiver’s depressive symptoms and perception of stress” Pinyopornpanish et al., 2021, p. 1).

The most widely used measure of perceived stress is the Perceived Stress Scale (Teresi et al., 2020), including in persons living with dementia and their caregivers (Deeken et al., 2018). It has been translated into several languages (Deeken et al., 2018; e.g., Spanish, Japanese, Arabic, Greek, Thai) and consists of 14 items. It is scored on a 5-point Likert scale, ranging from 0 (never) to 4 (very often); totals range from 0 to 40 with higher scores indicating higher perceived stress (Pinyopornpanish et al., 2021). An advantage of the PSS for persons with dementia lies with its brevity, which is an important consideration for persons with cognitive impairment (Deeken et al., 2018), who are often under the additional stress of undergoing testing outside the comfort of their home. Finally, the PSS will serve as a non-invasive measure of stress alternative to salivary cortisol.

#### 1.2.4 Design and procedure

This is a cross-sectional study design to examine the relationship between perceived stigma and perceived stress primarily. Concurrently, this study examined the effect of the HABIT program intervention on stigma and stress, in persons with dementia and their caregivers. The HABIT program served as the independent variable. Perceived stigma and perceived stress served as the dependent variables, measured by the SIS and PSS respectively, both pre and post intervention. Perceived stigma was used to predict perceived stress before and after completion of the HABIT program in this regard. Income was included as a covariate in the linear regression.

Once enrolled, and preceding the start of the group program, HABIT participants were provided the opportunity to consent in-person by the principal investigator. Upon arrival on the first day of the HABIT program, patients with MCI were administered the SIS and PSS, while the caregiver partners were administered the SIS-Caregiver version and PSS. The participants then proceeded with the 10-day treatment HABIT program as planned. After completion of the HABIT program, the measures were repeated on the last day. Outcome measures included levels of perceived stigma and perceived stress in persons with MCI and care partners.

#### 1.2.5 Data analysis

IBM SPSS Statistics version 27 was used for the data analysis. Descriptive statistics revealed a relatively high level of income for the study population, which is reported in Table 1. Mean test scores of all outcome measures were calculated pre- and post-HABIT intervention for the total and 2 groups separately. Two linear regression analyses were run to examine the relationship between perceived stigma and perceived stress before and after the HABIT program, one without covariates and another including income as a covariate. These results are summarized in Tables 2, 3.

**Table 1:** Income.

	Arizona			Florida		
Variable	N	Mean	SD (range)	N	Mean	SD (range)
Income	14	8.14	1.099	16	8.63	0.5

**Table 2:** Linear regression Pre and Post HABIT.

Variable	B	t	p	%CI
Pre-HABIT	0.122	1.113	0.275	[−0.102, 0.346]
Post-HABIT	0.432	3.201	0.003	[0.156, 0.709]
Pre-HABIT Social Rejection	0.200	0.648	0.522	[−0.432, 0.832]
Post-HABIT Social Rejection	0.449	1.386	0.177	[−0.214, 1.112]
Pre-HABIT Financial Insecurity	−0.529	0.595	0.557	[−2.351, 1.292]
Post-HABIT Financial Insecurity	0.449	1.386	0.177	[−0.214, 1.112]
Pre-HABIT Internalized Shame	0.200	0.138	0.891	[−0.766, 0.877]
Post-HABIT Internalized Shame	1.072	2.012	0.054	[−0.019, 2.163]
Pre-HABIT Social Isolation	0.538	2.363	0.025	[0.078, 1.089]
Post-HABIT Social Isolation	1.188	4.831	<0.001	[0.684, 1.691]

**Table 3:** Linear regression Pre and Post HABIT, controlling for income.

Variable	B	t	p	%CI
Pre-HABIT	0.128	1.288	0.209	[−0.076, 0.331]
Post-HABIT	0.442	3.445	0.002	[0.197, 0.705]
Pre-HABIT Social Rejection	0.298	1.064	0.297	[−0.277, 0.873]
Post-HABIT Social Rejection	0.532	1.699	0.101	[−0.110, 1.174]
Pre-HABIT Financial Insecurity	0.079	0.093	0.926	[−1.670, 1.829]
Post-HABIT Financial Insecurity	−0.238	−0.231	0.819	[−2.385, 1.882]
Pre-HABIT Internalized Shame	0.064	0.174	0.863	[−0.687, 0.814]
Post-HABIT Internalized Shame	0.972	1.841	0.077	[−0.111, 2.056]
Pre-HABIT Social Isolation	0.499	2.164	0.039	[0.026, 0.972]
Post-HABIT Social Isolation	1.152	4.838	<0.001	[0.664,1.641]

### 1.3 Results

Thirty participants were enrolled in Jacksonville, Florida and Scottsdale, AZ, consisting of 15 persons with MCI and 15 caregivers. In Florida, there were 14 participants (7 MCI-Caregiver dyads) and 16 participants (8 MCI-Caregiver dyads) in Arizona. There were an equal number of males and females, and all dyads were married. The mean age of participants with MCI was 74 years of age. The mean age of caregivers was 72.27. The mean age of the entire group was 73. All but two participants had at least a college level degree, while 19 participants had graduate or doctorate level degrees. Seven participants had an annual income between $100, 000 and $149, 000, and 18 participants had an annual income of $150,000 or more. Mean income for the total group was 8.4 (Scored 1–9) which is in the middle of the $100,000–$149,999 range. Due to an unexpectedly high level of income for the majority of participants, it was decided to include income as a covariate in the data analysis, especially considering the tendency for higher socioeconomic status to serve as a protective factor against stress (Schmitt et al., 2023; Table 1).

**Table 4:** PSS and SIS Scores of MCI and Caregiver Groups.

	Pre HABIT PSS Total	Post HABIT PSS Total	Pre HABIT SIS Total	Post HABIT SIS Total	Pre HABIT Social Rejection	Post HABIT Social Rejection	Pre HABIT Financial Insecurity	Post HABIT Financial Insecurity	Pre HABIT Internalized Shame	Post HABIT Internalized Shame	Pre HABIT Social Isolation	Pre HABIT Social Isolation
1	22	15	48	19	15	3	0	0	15	11	18	5
2	30	27	41	35	12	10	2	2	11	7	16	16
3	20	30	32	49	6	12	0	1	12	14	14	22
4	24	20	36	32	10	11	1	0	8	8	17	13
5	30	38	41	39	15	15	1	3	6	5	19	16
6	13	22	36	39	13	16	3	0	11	8	9	15
7	33	28	28	25	9	9	2	0	7	8	10	8
8	25	22	0	22	0	8	0	2	0	5	0	7
9	24	30	48	40	14	14	4	0	13	12	17	14
10	32	29	19	25	5	3	2	0	5	11	7	11
11	26	24	52	41	16	12	4	2	13	13	19	14
12	23	16	45	24	16	8	4	2	10	5	15	9
13	20	17	41	48	17	22	6	6	5	5	13	15
14	21	11	7	9	2	1	0	0	1	5	4	3
15	34	36	40	46	10	14	0	1	11	10	19	21

MCI group.

**Table 5:** Caregiver group.

	Pre HABIT PSS Total	Post HABIT PSS Total	Pre HABIT SIS Total	Post HABIT SIS Total	Pre HABIT Social Rejection	Post HABIT Social Rejection	Pre HABIT Financial Insecurity	Post HABIT Financial Insecurity	Pre HABIT Internalized Shame	Post HABIT Internalized Shame	Pre HABIT Social Isolation	Pre HABIT Social Isolation
1	4	2	22	21	8	8	2	2	5	5	7	6
2	14	23	34	29	10	10	5	3	10	7	9	9
3	21	16	28	29	9	11	0	0	11	9	8	9
4	9	9	27	27	7	8	2	2	9	10	9	7
5	13	13	35	28	13	10	0	3	11	6	11	9
6	13	14	12	24	4	8	0	0	7	9	1	7
7	21	27	30	24	9	3	3	0	10	11	8	10
8	21	18	29	30	13	13	2	1	7	10	7	6
9	27	25	5	20	0	5	0	0	2	7	3	8
10	15	18	24	24	9	9	3	3	5	5	7	7
11	38	16	35	22	12	5	2	1	7	7	14	9
12	29	29	45	35	15	11	2	2	12	10	16	12
13	26	20	47	29	17	15	3	4	11	8	16	12
14	16	15	23	23	8	8	3	3	5	5	7	7
15	27	28	27	20	8	6	2	1	8	6	9	7

The pre-measures were aimed to measure existing levels of perceived stigma and perceived stress in participants, and the post-measures were designed to measure levels of perceived stigma and stress after completion of the HABIT program to determine the effect of the intervention (Tables 4, 5). In the assessment of the average scores of perceived stigma and stress outcome measures (PSS and SIS), the total group, MCI group, and Caregiver group scores all decreased slightly from pre to post HABIT intervention (Pre HABIT PSS Total: *M* = 22.3667, *SD* = 7.85859; Post HABIT PSS Total: *M* = 21.1667, *SD* = 8.03477; Pre HABIT SIS Total: *M* = 31. 2,333, *SD* = 13.26828; Post HABIT SIS Total; *M* = 29. 6,000; *SD* = 9. 61,894; Pre HABIT PSS MCI: *M* = 25. 133, *SD* = 5.79244; Post HABIT PSS MCI: *M* = 24. 333, *SD* 7.77970; Pre HABIT SIS MCI: *M* = 34. 2,667, *SD* = 15. 06399; Post HABIT SIS MCI: *M* = 32. 8,667, *SD* 11. 77,689; Pre HABIT PSS Caregiver: *M* = 19. 6,000, *SD* = 8.83014; Post HABIT PSS Caregiver: *M* = 18.000, *SD* = 7.19126; Pre HABIT SIS Caregiver: *M* = 28.2000, *SD* = 10. 86,410; Post HABIT SIS Caregiver: *M* = 26. 333, *SD* = 5.48591). To determine if income had an influence on perceived stress and stigma scores, an independent samples T-test was performed and revealed it was not significant for pre-HABIT PSS total but was very close [*t*(28) = 2.029, *p* = 0.052]. It was not significant for pre-HABIT SIS total [*t*(28) = 1.265, *p* = 0.216] but was statistically significant for post-HABIT PSS scores between the 2 groups and the effect size was large as measured by Cohen’s d [*t*(28) = 2.315, *p* = 0.028*, d* = 0.845]. Finally, it was not significant for post-HABIT SIS total at [*t*(28) = 1.948, *p* = 0.062]. Commensurately, when looking at the confidence intervals, only the post HABIT PSS total score did not cross 0. A linear regression was then performed to examine the relationship between perceived stigma and perceived stress pre- and post-HABIT. Pre-HABIT Perceived stigma is not a significant predictor of pre-HABIT Perceived Stress (*B* = 0.122[−0.102, 0.346], *t* = 1.113, *p* = 0.275), but post-HABIT Perceived stigma is a significant predictor of post-HABIT Perceived Stress, (*B* = 0.432[0.156, 0.709], *t* = 3.201, *p* = 0.003). The same linear regression was performed that included the 4 SIS subscales to see if any of them were more contributory and found that only one subscale, the social isolation subscale, to be statistically significant both pre and post-HABIT [Pre-HABIT Perceived Stigma Social Isolation score is a significant predictor of pre-HABIT Perceived Stress, (*B* = 0.583[0.078, 1.089], *t* = 2.363, *p* = 0.025); post-HABIT Perceived Stigma Social Isolation score is a significant predictor of post-HABIT Perceived Stress, (*B* = 1.188[0.684, 1.691], *t* = 4.831, *p* < 0.001)]. It is worth noting that post-HABIT internalized shame score came very close to being statistically significant (post-HABIT Perceived Stigma Internalized Shame score is not a significant predictor of post-HABIT Perceived Stress, *B* = 0.1.072[−0.019, 2.163], *t* = 2.012, *p* = 0.054).

Finally, the linear regression was repeated with the additional control for income and found that adding income to the model provided better information to predict perceived stress both pre-HABIT [*F*(2,27) = 4.336, *p* = 0.023] and post-HABIT [*F*(2,27) = 7.734, *p* = 0.002]. The total sample linear regression controlling for income pre-HABIT found that total annual income was a significant predictor of pre-HABIT PSS Total Score, (*B* = −4.119[−7.277, −0.960], *t* = −2.676, *p* = 0.013). However, pre-HABIT SIS was not a significant predictor pre-HABIT PSS score (*B* = 0.128[−0.076, 0.331], *t* = 1.288, *p* = 0.209). The total sample linear regression controlling for income found that annual income was not a significant predictor of post-HABIT PSS Total Score, (*B* = −2.919[−5.880, 0.042], *t* = −2.203 *p* = 0.053). However, post-HABIT SIS was a significant predictor of perceived stress (*B* = 0.442[0.179, 0.705], *t* = 3.445, *p* = 0.002).

The total sample linear regression controlling for income for the SIS subscales found Social Rejection and Social Isolation to be the most significant predictors of perceived stress. Marginal significance was found for Financial Insecurity and Internalized Shame. The significant results are summarized as follows: The SIS Social Rejection, controlling for income pre-HABIT found the model adding income, provided better information to predict perceived stress, *F*(2,27) = 4.009, *p* = 0.030 and total annual income was a significant predictor of pre-HABIT PSS Total Score, *B* = −4.289[−7.503, −1.076], *t* = −2.739 *p* = 0.011. The SIS Financial Insecurity, controlling for income pre-HABIT found the total annual income was a significant predictor of pre-HABIT PSS Total Score, *B* = −4.120[−7.515, −0.725], *t* = −2.490, *p* = 0.019. The SIS Internalized Shame, controlling for income pre-HABIT found that total annual income was a significant predictor of pre-HABIT PSS Total Score, *B* = −4.078[−7.329, −0.826], *t* = 0.174 *p* = 0.016. The SIS Social Isolation, controlling for income pre-HABIT found the model adding income, provided better information to predict perceived stress, *F*(2,27) = 6.218, *p* = 0.006, and total annual income was a significant predictor of pre-HABIT PSS Total Score, *B* = −3.591[−6.629, −0.553], *t* = −2.425 *p* = 0.022. Pre HABIT SIS Social Isolation was also a significant predictor of perceived stress *B* = 0.499[0.026, 0.972], *t* = 2.164, *p* = 0.039. The SIS Social Isolation, controlling for income post-HABIT found the model adding income, provided better information to predict perceived stress, *F*(2,27) = 14.035, *p* < 0.001. However, post HABIT SIS Social Isolation was a significant predictor of perceived stress *B* = 1.152[0.664, 1.641], *t* = 4.838, *p* < 0.001.

### 1.4 Discussion

This study aimed to evaluate the relationship between perceived stigma and perceived stress in the early stage of MCI, the effect of income on levels of perceived stigma and stress, and whether a combined cognitive rehabilitation and wellness intervention would affect these perceptions. The small sample size notwithstanding, the results support the possibility of a relationship between perceived stigma and perceived stress, a relationship between income and levels of perceived stigma and stress, and that the HABIT program would affect this relationship. The HABIT intervention had an unanticipated effect than initially expected. The initial prediction expected the program to ameliorate levels of perceived stigma and stress, especially considering prior positive outcomes of improved memory and activities of daily living, heightened self-efficacy, improved mood and quality of life, and decreased anxiety (see Locke et al., 2021). These positive outcomes were initially expected to mitigate levels of perceived stigma and stress. However, the evidence demonstrated that on the whole, perceived stigma is a significant predictor of perceived stress *post* but not pre intervention), with the exception of the social isolation SIS subscale that was significant both pre-HABIT and post-HABIT. The longitudinal findings from Burgener et al. (2015) found that perceived stigma (measured by the SIS) was associated with several quality-of-life outcomes; including anxiety; depression; behavioral symptoms; health; personal control; self-esteem; social support understanding; and activity participation in persons with dementia and their caregivers. It’s important to note that they did not measure perceived stress specifically, although one may reasonably suspect that higher levels of stress are associated with these feelings and symptoms. It is possible that due to the very early stage of MCI, the participants and their caregivers were unaware of the immense difficulties and challenges that could be faced over time as the disease progresses. Ignorance may be bliss in this regard but perhaps not for the better. It would certainly account for the significance found post HABIT that was not present pre HABIT. Perhaps, through the educational and counseling components of HABIT, they became more aware of these challenges, their own self-perceptions and levels of stress, as well as the severity of their own limitations. Once the potential gravity of the diagnosis was realized, it is possible that their perceptions were consistent with prior studies supporting the fear, uncertainty, and stigma attached to the diagnosis of MCI (Beard and Neary, 2013; Bermejo-Pareja et al., 2016; Morris et al., 2020). In a study by Stites et al. (2017), persons with MCI or AD who were aware of their diagnosis reported lower satisfaction with daily life, basic functioning, and well-being, as well as more difficulties in daily life than those who were unaware. In this study, participants were aware of their diagnosis, but possibly not the implications thereof. What’s more, participants in the Stites et al. (2017) study who expected their condition to worsen reported higher levels of depression and stress, which may underscore the effect that education can have on perceived stress. Since the HABIT program was short duration (10 days), one can reasonably rule out the chance that any changes were the result of the natural course of MCI progression rather than recent intervention. Furthermore, for this particular group of participants, income was a significant predictor of perceived stress pre HABIT but not post HABIT. Considering the higher level of income of all participants, it came to no surprise that all participants scored low on the financial insecurity portion of the SIS. So, assuming income is a protective factor for perceived stress, again the intervention may have highlighted the gravity of the disease to the point that income no longer served as a protective factor for perceived stress after participants were educated about the challenges that may ensue.

Of the 4 SIS subscales. Social Isolation and Social Rejection appeared to have the most impactful element of stigma perception, and most significant relationship with perceived stress. One of the negative effects of stigma in persons with MCI and dementia and caregivers is increased social isolation, loss of social support, and feelings of social rejection (Lion et al., 2020; Rosin et al., 2020). These results seem consistent with the magnitude of social isolation and social rejection in this regard. Lion et al. (2020) found a similar relationship between perceived stigma (measured by the SIS) and social support, and emphasized the importance of social support as a modifiable factor in perceived stigma.

Taken together, the results of this small study suggest that a relationship exists between perceived stigma and perceived stress in persons with MCI, and the negative social consequences of stigma produced the most stress. It also infers that the more aware persons with MCI become with their diagnosis and the difficulties that may occur, the more the relationship between stigma and stress is strengthened. Higher income appears to be a protective factor for levels of perceived stigma and perceived stress in persons with MCI, but only up to a point. After learning about the challenges of the disorder, the strength of income as a protective factor appears to diminish. The is marked paucity of literature on perceived stress and MCI, as well as perceived stigma and MCI. At the time of this writing, no literature was found on the relationship between perceived stigma and perceived stress in person with MCI to either support or negate these findings.

The limitations of this study were vast and primarily products of research design, reliance on the subjectivity of self-report, small sample size, and limited resources. First and foremost, this was a convenience sample that was under powered due to the small sample size. Furthermore, the study design along with resource limitations did not allow for random selection or a waitlist control group. While the employed measures are essentially the gold standard for perceived stress and stigma, there remains the subjectivity of self-report inherent in any questionnaire. Persons with early phase cognitive decline, such as the case of MCI, may not yet be familiar with the full experience of stigma and associated stress that may arise. Importantly, there was no opportunity of comparison to early and late AD to the MCI group to evaluate to what extent cognitive impairment affects perceived stigma and perceived stress, which would be ideal in a follow-up study.

Persons with MCI, dementia, and caregivers who feel stigmatized are more likely to avoid cognitive evaluation, delay diagnosis, and tend to isolate themselves, which would infer they would not be the type of population seeking out an intervention such as the HABIT program. Additionally, the chosen convenience population may have had a pre-existing bias considering their levels of motivation, income, and social support necessary for enrollment in the HABIT program. Those who are able to enroll in the HABIT program are likely to be highly motivated with access to good health care and medical referrals. Speculation notwithstanding, the very reputation and prestige of the Mayo Clinic may lead to a more positive attitude on the part of participants. The timing and biases inherent in the study, therefore, may not be entirely representative of the population.

Lastly, appropriate measures of stigma and the stress response have not yet been developed. An approach to evaluate the reverse causality of perceived stress on perceived stigma is also needed. The SIS is the most widely used measure of perceived stigma, but it is important to remember that there is no “gold standard” at the time of this study. Additionally, using the PSS, while advantageous in its ease of use for those who have cognitive difficulties, may be further strengthened by a biomarker of the chronic stress response, which salivary cortisol would capture, but for the purpose of this study the PSS was intended to be less invasive and onerous to the participants who are already undergoing a battery of testing. Furthermore, a confounding variable regarding the source (s) of stress is associated with using the PSS, despite its advantages of brevity and accuracy in general, it is not targeted to measure stress associated with dementia or stigma specifically. Long-term, it would be interesting to know if the perceived stress derived from perceived stigma actually accelerates cognitive decline, and thus affects the fate of progression from MCI to AD. Ideally, a future study will incorporate biomarkers of the physiological stress response including salivary cortisol, hs-CRP, TFN-α, and IL-6 (Milligan Armstrong et al., 2021) in addition to perceived stigma and perceived stress measures.

## 2 Conclusion

While approximately 50 million people worldwide are living with Alzheimer’s disease (Herrmann et al., 2018), many more are living with Mild Cognitive Impairment (MCI), a condition that often represented as the earliest phase of the disease (Breton et al., 2019). Among the aging population in general and persons with MCI, fear of developing AD is a common concern (Norman et al., 2020), perhaps in part due to this representation of AD’s prodromal phase. In addition to financial burdens, persons with dementia and caregivers experience heavy tolls of emotional distress, negative physical outcomes, and detrimental psychological effects (Alzheimer’s Association, 2023). Such tolls are not restricted to those currently affected by dementia, but also include those with MCI who live in fear of developing dementia (Beard and Neary, 2013) along with their care partners (Carlozzi et al., 2018).

There appears to be a relationship between perceived stigma and perceived stress in persons with MCI and their care partners. The label of MCI may contribute a substantial amount to these perceptions, and therefore carry heavy implications and cautions for its use among healthcare providers. “What’s in a name” is quite far reaching in this regard. This is not to say the diagnosis should be avoided entirely, but judiciously. When warranted, diligent efforts to educate patients and caregivers regarding the rates of regression and progression may, at least in part, reduce the fear associated with the diagnosis of MCI. The stigma surrounding AD and dementia in general will necessitate interventions on several levels to educate individuals, create a culture change, and shift societal perceptions. The media, of course, would also need to participate by cessation of its current approach of depicting persons with dementia in the worst possible scenarios.

For persons with MCI and their care partners, it’s worth noting that education may have a different effect on their perceptions. In this study, the HABIT program intervention surprisingly appeared to strengthen the relationship between perceived stigma and stress, rather than decrease levels of perceived stigma and stress, which may not carry a negative connotation. A positive corollary to this effect may in turn aid in the opportunity for patients and caregivers to seek appropriate care, make appropriate life plans, and obtain social support. Providing persons at higher risk for dementia, as with the case of MCI, with tools and informed insight may facilitate the successful navigation of their journey. More research is needed to substantiate these relationships and recommendations, ideally with a larger sample size, comparison groups, and biomarker evaluation.

Certainly, cognitive impairment of any type is devastating for those who both experience and witness its progression. Persons with dementia, persons with MCI at higher risk for dementia, and caregivers should not have to endure the additional burden of stigma attached to these diagnoses, but rather be supported. Stigma is not only an impedance to dignity and humane treatment but is also an obstacle for persons with MCI to take advantage of interventions that may decrease their likelihood of progression. Hopefully this is one miniscule step to understanding their experience and working toward eradication of dementia-related stigma.

## Data availability statement

The datasets presented in this article are not readily available because restricted by Harvard University IRB. Requests to access the datasets should be directed to aliwarren@gwu.edu.

## Ethics statement

The studies involving humans were approved by Harvard University IRB/Harvard University Committee on the Use of Human Subjects. The studies were conducted in accordance with the local legislation and institutional requirements. The participants provided their written informed consent to participate in this study.

## Author contributions

AW: Writing – original draft.
